# Income, wealth and use of personal protection equipment in the Mekong Delta

**DOI:** 10.1007/s11356-021-13449-w

**Published:** 2021-03-25

**Authors:** Matteo Migheli

**Affiliations:** Department of Economics and Statistics “Cognetti de Martiis”, University of Torino, Lungo Dora Siena 100, I-10153 Torino, Italy

**Keywords:** Personal protective equipment, Agrochemicals, Income, Wealth, Vietnam

## Abstract

Both in developing and developed countries, farmers often do not protect themselves adequately, especially when applying agrochemicals that are dangerous for their health. The issue is relevant because insufficient protection is between the causes leading to intoxication of farmers and workers who handle these products. The literature suggests that both lack of training and information and low income may explain why, especially in developing countries, protective equipment is under-used. Using data from the Mekong Delta, this study addresses the issue of whether income and household wealth may help explaining the use of incomplete protections against pesticides. The results suggest that income, more than wealth, is a reason why Vietnamese farmers operating in the Mekong Delta fail in using adequate protections. In particular, the data suggest that they may prefer to divert resources to increasing the production of their fields or to buying goods that may be used both as protection and as everyday garments. This behaviour leads to underinvestment in some important protective goods. Possible public interventions to mitigate the problem are suggested; in particular, the promotion of integrated pest management techniques could be useful.

**Supplementary Information:**

The online version contains supplementary material available at 10.1007/s11356-021-13449-w.

## Introduction

“Over 150,000 people die each year from pesticide poisoning. […] Severe pesticide poisoning is more common in rural lower- and middle-income countries where pesticides are widely used in smallholder agricultural practice”.^1^ Poisoning due to agrochemicals pertains to the broader set of sustainability (Salas-Zapata and Salas-Zapata 2017); pesticides may indeed cause acute and chronic illnesses ranging from cancer to reproductive abnormalities (Kasiotis et al. 2012). Farmers and their children are often subject to exposure to pesticides and display symptoms of poisoning, with no apparent improvement during the last decades (Rubino et al. 2012). However, casualties of intoxication from pesticides occur among farmers in developing countries because of lack of money to purchase PPE and insufficient training and information on both the use of agrochemicals and protections (Marcus and De Souza 2020).

There are several reasons for farmers being poisoned. On the one hand, they use pesticides and work in fields treated with agrochemicals. They absorb these through inhalation and through contact with the skin. On the other hand, besides inevitable accidents, farmers often do not follow usage instructions and/or do not wear (appropriate) PPE, especially in developing countries (Dinham 2003 and Karunamoorthi et al. 2011). Moreover, sometimes the PPE used is old and damaged or simply inappropriate, as it is permeable to some specific chemicals used by the farmers (Reed et al. 2006). However, in these countries, people living in the countryside are very often illiterate and therefore cannot read the instructions for the correct use of agrochemicals (Dinham 2003), and farmers who know the risks for their health often do not use adequate PPE (Blanco-Muñoz and Lacasaña 2011), because their socio-economic conditions do not allow them to buy sufficient PPE (Rahman 2015). According to the existing works, however, the main problem is not the absence of use of PPE; rather, farmers and agricultural workers in general are found to use some protective devices but inadequately. Such a behaviour suggests that people are aware of the risks (otherwise they would not use any protection), but do not take all the recommended and necessary precautions.

The aim of the present paper is to understand to which extent the economic situation of farmers in the Mekong Delta affects their use of PPE. As the following section will show, there is scarce literature on the use of PPE in Vietnam. Therefore, this paper also contributes evidence from an area—the Mekong Delta—with a very high concentration of agricultural activities and scarce coverage of research in the field of PPE. In particular, the empirical analysis focuses on both their income and the wealth of their household. Indeed, the behaviour of pesticide users is crucial to prevent cases of poisoning both among farmers and the consumers of the agricultural products (Damalas and Koutroubas 2018). The analysis focuses on small farmers working in the Mekong Delta. This region is relevant for several reasons. First, the region has a very high density of farmers, producing most of the Vietnamese rice and making this country the second largest exporter of rice in the world, according to the FAO. Given this large production of rice, the area is well known for the intensive use of pesticides (Houbraken et al. 2016). Second, the use of pesticides in paddies is particularly harmful, as the farmers often work with their feet and legs in the water, in which high concentrations of pesticides are present. Therefore, it seems that rice fields expose the workers to more sources of contamination than other cultivation methods. Third, Vietnam has been very active in promoting integrated pest management and campaigns to sensitise the farmers about the risks for their health due to the use agrochemicals (Dang et al. 2017). Fourth, the current distribution of land in the Mekong Delta is largely the consequence of the *Doi Moi* (literarily “renovation”) policy of transition from collectives to private farms actuated during the last two decades (Migheli 2012). This is relevant from a policy point of view. Indeed, if this redistribution has endowed households with insufficient land to produce enough income to afford PPE, then the public authorities should intervene by either revising the redistribution operated or subsidising the farmers.

## Literature review

Most of the extant studies on the use of PPE among farmers in developing countries focus on two main issues: awareness and—related to it—participation in specific educational programmes. However, the fact that the workers who are aware of the risks of using pesticides also do not use PPE is somehow puzzling. Indeed, one should expect people to wish to protect their health above all. Of course, it is known that people deliberately use products that are harmful to their health (e.g. tobacco and alcohol), but in these cases, the potential damage is (partially) offset by the utility deriving from consuming these products. Instead, the toxicity of pesticides is not balanced by any personal utility, as in the case of tobacco and alcohol. Few studies address the problem of the socio-economic conditions of the farmers. Indeed, PPE is relatively expensive, especially if it is of good quality. The present work aims to inquire into the association between households’ income and wealth and the use of PPE. In particular, the level of PPE, the expenses for purchasing PPE, and its condition constitute the main variables of interest.

Among the pillars of the *Doi Moi* were the privatisation of the agricultural sector and the liberalisation of the input market; this step in the transition from a planned to a market economy boosted the agricultural production, especially in the very fertile region of the Mekong Delta (Roy 2016). A large increase in the use of agrochemicals and—in particular—of pesticides accompanied the terrific increase in production (Toan et al. 2013). Some studies show that farmers use pesticides to increase the productivity of their fields for two reasons: first, they aim to maximise the income from their farming activity and, second, the increases in production obtained allow them to work part of their time outside the farm, gaining additional income.

Migheli (2017) and Sapbamrer and Thammachai (2020) show that a major problem common to other developing countries is that as in this part of Vietnam, farmers apply also pesticides—such as DDT—that are officially forbidden by the national law. Migheli (2017) also reports high levels of intoxication, as a consequence of both the use of dangerous products and poor personal protection. The literature on the use of PPE in Vietnam is scarce; however, the extant works show that the situation is very similar to that in most other countries.

The literature on the use of PPE when farmers use agrochemicals is wide; the present review will summarise a synthesis of the most recent contributions related to research question. Sapbamrer and Thammachai (2020) show that the underuse of PPE is diffused worldwide and characterises both developing and developed countries. In their systematic review, the two authors identify several socio-demographic causes of such a misuse. In Europe, a large share of Greek farmers (49.3%) do not use adequate PPE, especially when they are old and have never been empoisoned by pesticides (Damalas and Abdollahzadeh 2016), as they perceive low risks related to the use of pesticides (Damalas et al. 2019). Rezaei et al. (2019) consider a sample of Iranian cultivators and find similar results: Risk perceptions and past poisoning experiences drive the choice of adopting PPE.

The situation in some low-income countries, such as Ghana, is even worse: Okoffo et al. (2016) show that about one-fifth of the farmers in that country do not wear any PPE, while the most of the others protect themselves only partially. Ethiopian farmers use adequate PPE only in 10% of cases, and 62% of them do not take a shower after applying pesticides, even when using scarce protections (Negatu et al. 2016). Houbraken et al. (2017) find similar misuse of pesticides in Rwanda, where farmers use large quantities of them, poisoning the surface of lakes. Kwakye et al. (2019) show that also the level of farmers’ education plays a relevant role in increasing the use of PPE in Ghana, likely because users of pesticides and agrochemicals have serious difficulties in reading and understanding the labels of the products and the suggested precautions (Dugger-Webster and Le Prevost 2018). Similar results are also found in the case of Kuwait, a country with higher per capita income than Ghana and Ethiopia (Jallow et al. 2017).

The most of the existing studies on the behaviour of farmers using agrochemicals have focused on the perception of the risks related to the use of such products, with little or no attention paid to the impact of the economic conditions of the households on the use of PPE. While this lack of protection is sometimes the result of unavailable information or inadequate training (Lopez-Davila et al. 2020 and Sapbamrer and Thammachai 2020), Migheli (2020) and Naveed and Hassan (2020) highlight that also specific training and informative campaigns may be insufficiently effective. In particular, the results of Kwakye et al. (2019) suggest that the economic conditions of the households—proxied by education—may matter. Mubushar et al. (2019) analyse a sample of Pakistani farmers and inquire the relationship existing between their level of education and land ownership (captured by a dummy variable) on the one side and the safety in the use of pesticides on the other. The authors find a positive correlation between the first two variables, used as regressors, and the third, used as dependent variable in the regression. Once more, this result suggests that not only the level of education of the farmers but also the economic conditions of their households are related to affecting how they handle agrochemicals and how they protect themselves from these products.

With specific reference to the Vietnamese case, Thi Thuy et al. (2012) find that Vietnamese farmers are generally unaware of the real risks connected to the use of pesticides and therefore use inadequate PPE and apply dangerous chemicals, often incorrectly. As early as Dung and Dung 1999, Dung and Dung observe that Vietnamese farmers do not adopt sufficient precautions to protect themselves from agrochemicals. However, in spite of the efforts of the government to sensitise farmers about the risks associated with the use of pesticides, the situation has not changed very much over time. Phung et al. (2012) report that the use of adequate PPE in the Mekong Delta is very rare, in particular because farmers often wear incomplete protection. Phung et al. (2013) stress once more that several workers in Vietnamese paddies, especially those situated in the Mekong Delta, often wear shirts and trousers but do not wear gloves, glasses and masks to prevent inhalation and absorption through the nasal mucosa and the eyes.

To face the increasing use of pesticides, the Vietnamese Government started educational campaigns aimed at informing farmers about the risks related to the use of these chemicals and about the adoptable measures to decrease these risks. These campaigns focused on integrated pest management practices and on the use of PPE. Unfortunately, the fragmentation of rural property, which followed the early policies of transition, made these programmes largely ineffective (McCann 2005). Some policies aimed at rationalising the seeding procedures (i.e. reducing the quantity of seeds per hectare) had the effect of decreasing the use of agrochemicals (including pesticides) while improving the yields; however, given the counterintuitive nature of the phenomenon, these measures did not meet the farmers’ favour and had very limited success (Huan et al. 2005).

## Data and methodology

The data used in this paper are from the World Bank. A survey covering 603 farmer households^2^ in the Mekong Delta contains information about the following:

- Socio-economic conditions of these households (in particular their income, its sources and the ownership of several assets)
- Use of pesticides
- Use, cost and conditions of PPE worn during work
- Crop yields in the past two cropping seasons, the prices of the main crops
- Other relevant variables, such as the extension of the cultivated land, the perceived harmfulness of the pesticides used, their concentration and the equipment used to spray them.

The website of the World Bank clarifies that “The survey was constructed by the World Bank team in collaboration with the University of Economics in Ho Chi Minh City (for socio-economic portion of the survey) and the Centre of Occupational and Environmental Health (COEH) of the Vietnam Association of Occupational Health (VINOH) for the medical survey. Each of these World Bank counterparts was responsible for the implementation of the survey. To minimize any possible reporting bias, the survey was conducted under the agreement that the team would not reveal the identity of the farmers surveyed or the respondents”.

The main variables of interest are those capturing the “level” of PPE and the income and the wealth of the household. The first is measured using two types of variables. On the one hand, the annual expenditure of the household on PPE provides information about the total investment in protection. As the number of people living in a household varies, the per capita value is used instead of the total amount spent. On the other hand, the quantity of PPE is assessed considering which form of protection (i.e. shirts, gloves, trousers, masks, glasses) the farmers use when preparing and spraying pesticides and washing the equipment used for it. This level of protection is assessed by reducing the different protective devices to a couple of continuous variables through principal component analysis. Table 1 shows the variables that enter the analysis and the correlations between each variable included in the analysis and the components extracted using the principal component analysis (PCA). In particular, as suggested by the extant literature, only those components whose eigenvector exceeds 1 are retained and named “component 1” and “component 2”, which are orthogonal to each other by construction. The procedure produces two components characterised by an eigenvalue greater than one; these components are retained.

**Table 1:** Correlations between the two components measuring PPE and the equipments

	Component 1	Component 2
Shoes	− 0.200***	− 0.397***
Hat	− 0.367***	− 0.436***
Glasses	− 0.465***	− 0.553***
Mask	− 0.573***	− 0.214***
Full sleeve shirt	− 0.637***	0.598***
Full length trousers	− 0.671***	0.578***
Gloves	− 0.483***	− 0.301***

***p < 0.01, **p < 0.05, *p < 0.1

The reason to use the components extracted through the PCA is that the literature does not provide any guide to compare the completeness and the efficacy of different partial protections. This means that it is not possible to state whether a set of gloves and trousers is more or less protective than a set of mask and shoes. Therefore, as ordering different sets is impossible or too much arbitrary, the values of the principal components, which are based on the explained variance of the underlying set of variables, are the least arbitrary measure of completeness of PPE.

Independent variables are introduced in the regressions on the basis of what the theoretical and empirical literatures suggest. On the one side, consumption depends on income and wealth, which are also the variables of interest for the analysis presented here. The completeness of PPE depends also on the quantity of agrochemicals used; in particular, if a farmer uses them seldom or on a very small plot, the risk of intoxication is lower than if chemical products are used on large plots and/or frequently. Consequently, farmers that fall in the first case may decide to minimise their PPE. Therefore, the extension of the cultivated land is used as a control. For the same reason, the frequency of use and the concentration of the products are introduced in the regressions. Different crops may require different quantities of agrochemicals and pesticides in particular; this is the reason why information about the extension of grown crops is used as a control variable.

The extant literature (see Section 2) suggests that a correlation exists between farmer’s education and the quality and completeness of PPE; therefore, the highest level of education attained by the farmer is used as a control. Finally, the perception about the riskiness of the products used is introduced in some specifications, as the level pf protection may depend on this variable. In the end, the full estimated model reads as follows:

$$ {PPE}_i={\beta}_0+{\beta}_1{I}_h+\gamma {\boldsymbol{W}}_h+\vartheta {\boldsymbol{L}}_h+\mu {\boldsymbol{P}}_h+\rho {E}_i+{\varepsilon}_i+{\tau}_h $$

where *PPE*_*i*_ is the level of PPE used by farmer *i* as measured by the components extracted through the PCA; *I*_*h*_ is the income at household level *h*; ***W***_*h*_ is the matrix of variables capturing household *h*’s wealth; and ***L***_*h*_ is the set of controls that include variables related to land ownership, extension and crops. ***P***_*h*_ is the set of pesticide-related controls; this variable is measured as a household level, as pesticides are applied to the land cultivated by the members of the household. *E*_*i*_ represents the farmer’s level of education. The Greek letters are the parameters to be estimated and include the error terms *ε*_*i*_ (individual level) and *τ*_*h*_ (household level).

The choice of the variables to include as independent elements in the estimated equation depends on both the theoretical suggestions (demand is a function of income and wealth) and on the extant empirical literature on pesticide use. In particular, the level of education and the size of land cultivated are generally found to be relevant explanatory variables of pesticide and PPE use.

The inclusion of expenditure on PPE that appears in some specifications may seem puzzling. However, its inclusion is necessary because of the definition of the dependent variable: Items that constitute the set of protections are different, and therefore they may have different prices. As it is not possible to order incomplete sets according to their degree of completeness, the quantity of money spent on them may help shedding additional light on the issue. In particular, a statistically non-significant coefficient would put forward that farmers are able to mix protections in an equally effective way independently on how much they spend on it. Such a result would be relevant, as it would suggest that training campaigns may be very effective if they teach the farmers how to choose the most effective sets of protections without increasing expenditure. While this would not render PPE complete when they are not, it would nevertheless increase the efficacy of protections. Unfortunately, as the results of the analysis will show, such a hypothesis is rejected by the data. All the empirical analyses presented in this paper were executed using STATA 15.0.

As Table 1 shows, the first component correlates negatively with all the forms of protection, while the second correlates negatively with all but trousers and shirts. For the sake of easing the interpretation of the results obtained in the next analyses, the two components are multiplied by − 1, so the first component represents a summative measure of all the possible protective devices, while the second component excludes—in a sense—trousers and shirts. These two components are used separately as dependent variables in the regression analyses. Furthermore, ordinary least squares (OLS) regressions are run for the amounts spent on each different form of protection listed and used by the farmers. In addition, the number of pesticide applications and the toxicity perceived by the farmers are used as control variables: the first because as the number of applications increases, so does the risk of health damage (Mage et al. 2000) and the second as risk awareness may have some influence on the farmers’ behaviour, although—as already stressed—the extant literature does not seem to find any relationship between awareness and the use of PPE.

For each dependent variable, regressions are run using six different specifications for the sake of testing the robustness of the main results. The regressors include income, wealth, number of household members, exposure to sensitising campaigns (dummy: 1 = yes, 0 = no), crop yields and prices in the two seasons preceding that in which the interview was performed. Households’ wealth is approximated by the ownership of durable goods, such as TV sets, radios, refrigerators and motorbikes. The ownership for a total of eighteen such goods is recorded, producing a set of eighteen dummies. To reduce the regressors to a reasonable number, I also run a principal component analysis on these eighteen dummies, retaining five components that capture the household wealth of the interviewees. Table 2 shows the Pearson correlation coefficients between each of the eighteen dummies and the five components retained.

**Table 2:** Correlations between indicators of household’s wealth and their principal components. *p* values in brackets

	Component 1	Component 2	Component 3	Component 4	Component 5
Television	0.425*** (0.000)	0.423*** (0.000)	0.045 (0.245)	− 0.015 (0.715)	0.022 (0.582)
Radio	0.423*** (0.000)	0.102** (0.012)	− 0.040 (0.328)	− 0.026 (0.522)	0.461*** (0.000)
Music player system	0.540*** (0.000)	− 0.017 (0.673)**	0.320*** (0.000)	0.234*** (0.000)	0.118*** (0.004)
Video/DVCD	0.664*** (0.000)	0.014 (0.734)	0.252*** (0.000)	0.295*** (0.000)	0.000 (0.996)
Bicycle	0.233*** (0.000)	0.439*** (0.000)	− 0.343*** (0.000)	0.142*** (0.000)	− 0.176*** (0.000)
Motorbike	0.645*** (0.000)	− 0.107*** (0.008)	− 0.193*** (0.000)	0.170*** (0.000)	− 0.063 (0.121)
Refrigerator	0.413*** (0.000)	− 0.377*** (0.000)	0.114*** (0.005)	− 0.066 (0.106)	− 0.395*** (0.000)
Electric fan	0.460*** (0.000)	0.471*** (0.000)	− 0.211*** (0.000)	− 0.185*** (0.000)	0.116*** (0.004)
Telephone	0.482*** (0.000)	− 0.355*** (0.000)	− 0.052 (0.198)	−0.038 (0.353)	− 0.297*** (0.000)
Computer	0.014 (0.730)	0.207*** (0.000)	0.238*** (0.000)	0.750*** (0.000)	0.003 (0.935)
Cooker	0.580*** (0.000)	0.212*** (0.000)	0.018 (0.662)	− 0.350*** (0.000)	− 0.092** (0.024)
Gas stove	0.662*** (0.000)	0.029 (0.476)	0.178*** (0.000)	− 0.216*** (0.000)	− 0.138*** (0.000)
Washing machine	0.133*** (0.001)	− 0.341*** (0.000)	0.301*** (0.000)	− 0.141*** (0.000)	0.168*** (0.000)
Bathroom/toilet	0.510*** (0.000)	− 0.149*** (0.002)	− 0.157*** (0.000)	0.032 (0.433)	− 0.157*** (0.000)
Motor boat	− 0.052 (0.199)	0.316*** (0.000)	0.689*** (0.000)	− 0.283 (0.000)	0.075* (0.067)
High quality furniture	0.519*** (0.000)	− 0.215*** (0.000)	0.034 (0.407)	− 0.038 (0.353)	0.415*** (0.000)
Pipewater connection	0.333*** (0.000)	− 0.125*** (0.000)	− 0.409*** (0.000)	− 0.030 (0.460)	0.496*** (0.000)
Other high value items	0.198*** (0.000)	− 0.037 (0.369)	0.006 (0.882)	− 0.040 (0.324)	0.022 (0.596)

***p value ≤ 0.01; ** 0.01 < *p* value ≤ 0.05; 0.05 < *p* value ≤ 0.1

Some works highlight the existence of a link between the gender of the farmer and the level of PPE used (Atreya 2007). Unfortunately, the survey used in this paper contains information almost exclusively for male interviewees (596 out of 603). Moreover, no information about the gender composition of the households is available. Consequently, the regressions do not include gender as a control.

Table 3 presents the descriptive statistics for all the variables used in the analyses. The figures in the table emphasise that farmers in the Mekong Delta do not use adequate PPE. In addition, in line with the extant literature, there is substantial variability with respect to the protection used: Only 2.5% of the interviewees wear shoes when applying pesticides, while almost all wear shirts and trousers (96.5% and 95.4%, respectively). While the available data do not include the prices of the single items that compose a complete PPE set, using data from the UNDP-GEF^3^, it is possible to calculate that farmers who score higher in protection completeness in the survey used in this paper spent about 6% of their yearly income on PPE.

**Table 3:** Descriptive statistics: means and standard deviations (in brackets)

Amount spent in PPE (VND, constant 2003)	41,050.58	Use of land (hectares)	
	(23,595.24)	Rice	1.267
Level of protection (principal components)			(1.199)
Component 1	0.000	Orchard	0.144
	(1.348)		(0.276)
Component 2	0.000	Aquaculture	0.008
	(1.217)		(0.046)
PPE use (dummies: 1 = yes)		Other crops	0.051
Shoes	0.025		(0.192)
	(0.156)	Yield of the main crop in the previous season	4.446
Hat	0.499		(1.112)
	(0.500)	Yield of the main crop in the second-to-last season	2.109
Glasses	0.199		(3.032)
	(0.400)	Price of the main crop in the previous season	752.65
Mask	0.633		(907.76)
	(0.482)	Price of the main crop in the second-to-last season	1609.10
Full sleeve shirt	0.965		(778.47)
	(0.183)	No education (dummy: 1 = yes)	0.013
Full length trousers	0.954		(0.114)
	(0.211)	Primary education (dummy: 1 = yes)	0.350
Gloves	0.179		(0.477)
	(0.384)	Secondary education (dummy: 1 = yes)	0.443
Number of applications (current season)	5.041		(0.497)
	(1.396)	Practices IPM (dummy: 1 = yes)	0.650
Cultivated land (ha)	1.471		(0.477)
	(1.259)	Formal training in IPM (dummy: 1 = yes)	0.517
Concentration of chemicals sprayed (mg/ha)	70.544		(0.500)
	(184.527)	Household wealth (principal components)	
Number of sprayers	0.814	Component 1	0.000
	(0.635)		(1.901)
Cost of sprayers	320,127.1	Component 2	0.000
	(392,278.3)		(1.128)
Amount borrowed (million dongs)	5.063	Component 3	0.000
	(10.412)		(1.110)
Income from remittances (dongs)	461,857.4	Component 4	0.000
	(2,144,181)		(1.033)
Per capita income (million dongs)	3.747	Component 5	0.000
	(3.824)		(1.012)
Income from crops (% of household income)	69.578		
	(27.946)		

## Results

Table 4 presents the OLS estimates, in which the dependent variable is the total expenditure on PPE at the household level. Higher levels of per capita income correspond to higher expenditure on PPE. In addition, higher prices of the main crop grown by the household in the second-to-last season also correlate positively with the expenditure on PPE. These two outcomes taken together suggest that farmers in the Mekong Delta do not use adequate PPE because they cannot afford it or, rather, because they prefer to use the money to extend their production instead of protecting themselves. Unfortunately, the available data do not allow the testing of this last hypothesis. Focusing on wealth, the figures suggest that the wealthier the household, the less it invests in PPE. Indeed, the coefficient of the extension of the land cultivated and the coefficients of the two principal components capturing wealth are negative and statistically significant at conventional levels for small samples (see the notes to the tables for further information). Only the coefficient of the second principal component of wealth is positive and statistically significant. Table 2 shows that the main difference between it and components 1 and 5 (which present positive and statistically significant coefficients) relates to transportation means and to home electrical appliances. However, given the opposite signs of the correlations between the principal components and the originating variables and the signs of the coefficients in the regressions, the message conveyed by all three principal components is the same. Therefore, the analysis of the principal components of wealth that are statistically significant suggests an overall negative effect of wealth on expenditure on PPE. While this result may appear to be counterintuitive, it may simply suggest that the farmers prefer to spend money on electric appliances and transportation means than on PPE. These goods improve the quality of life of these people immediately after their purchase, while the negative effects of intoxication are much less visible in the short run and the acute symptoms are temporary: PPE and the goods used to assess the households’ wealth seem to be substitutes.

**Table 4:** Determinants of expenditure for purchasing PPE

	1	2	3	4	5	6	7
Income per capita (million dongs)		1286 (247.7)***	1047 (219.5)***	1098 (233.2)***	987.2 (222.7)***	1032 (222.7)***	952.5 (222.6)***
Income from crops (% of total household income)		4.934 (32.91)	− 2.923 (31.66)	− 2.060 (31.63)	− 0.730 (31.60)	3.468 (31.56)	− 0.604 (31.68)
Remittances (dongs)		− 0.000267 (0.000229)	− 0.000608 (0.000281)**	− 0.000588 (0.000282)**	− 0.000614 (0.000282)**	− 0.000601 (0.000264)**	− 0.000621 (0.000291)**
Household’s wealth (principal components)							
Component 1			− 3044 (551.6)***	− 2911 (559.9)***	− 2341 (557.2)***	− 2513 (577.1)***	− 2398 (565.2)***
Component 2			1599 (781.1)**	1682 (784.0)**	1422 (753.3)*	1659 (769.4)**	1463 (757.8)*
Component 3			158.9 (869.2)	361.1 (854.9)	169.9 (858.5)	253.4 (868.9)	193.3 (877.2)
Component 4			− 247.0 (840.3)	− 317.7 (835.5)	− 419.4 (837.5)	− 363.9 (876.2)	− 444.4 (862.0)
Component 5			− 3407 (891.6)***	− 3153 (898.8)***	− 2688 (885.6)***	− 2757 (884.9)***	− 2714 (879.1)***
Extension of cultivated land (ha)	− 203.8 (823.9)	128.0 (887.7)	− 889.1 (932.2)	− 21,029 (12,382)*	− 35,238 (16,705)**	− 36,050 (13,809)***	− 38,690 (17,721)**
Number of pesticide applications in the current season	1480 (695.5)**	1358 (680.1)**	737.1 (618.0)	804.9 (617.4)	891.9 (617.8)	857.6 (625.7)	897.7 (626.8)
Concentration of pesticides sprayed (mg/ha)	− 9.125 (2.801)***	− 10.30 (2.639)***	− 9.399 (2.952)***	− 8.838 (2.882)***	− 8.218 (2.989)***	− 8.384 (2.992)***	− 7.774 (3.039)**
Number of pesticide sprayers		634.0 (2078)	− 184.6 (1908)	− 885.9 (1725)	− 875.0 (1712)	− 899.3 (1751)	− 879.5 (1723)
Average unit cost of sprayers (dongs)		− 0.00674 (0.00281)**	− 0.00659 (0.00218)***	− 0.00582 (0.00190)***	− 0.00577 (0.00199)***	− 0.00554 (0.00198)***	− 0.00553 (0.00201)***
Money borrowed (million dongs)		− 182.9 (87.07)**	− 195.6 (74.59)***	− 216.5 (65.57)***	− 224.7 (66.37)***	− 213.8 (66.35)***	− 229.7 (66.89)***
Age				− 35.05 (76.09)	23.90 (77.38)	− 21.17 (79.72)	14.06 (79.79)
Use of land (ha allocated to the following crops/activities)^1^							
Rice				19,816 (12,494)	34,356 (16,796)**	35,176 (13,936)**	37,736 (17,796)**
Orchards				29,133 (12,669)**	42,292 (16,954)**	43,016 (14,015)***	45,679 (17,947)**
Other vegetables				18,274 (13,077)	33,461 (17,101)*	34,040 (14,536)**	36,880 (18,144)**
Aquaculture				50,440 (26,694)*	61,019 (29,041)**	68,313 (27,216)**	64,681 (29,560)**
Yield of the main crop in the last season (q/ha)					378.8 (358.0)	500.6 (339.8)	364.6 (349.6)
Yield of the main crop in the second-to-last season (q/ha)					46.85 (79.56)	41.03 (85.60)	48.97 (79.09)
Price of the main crop in the previous season (đ/q)					1.605 (1.301)	1.390 (1.295)	1.564 (1.298)
Price of the main crop in the second-to-last season (đ/q)					3.282 (0.668)***	3.483 (0.731)***	3.270 (0.689)***
Practices IPM (dummy: 1 = yes)					3264 (1812)*		3280 (1837)*
Received formal training on IPM (dummy: 1 = yes)					3164 (1752)*		3158 (1768)*
Education							
No school						− 2915 (5478)	− 2551 (5196)
Primary school						792.8 (2681)	1476 (2745)
Secondary school						3038 (2544)	2988 (2578)
Level of risk perceived					339.2 (773.4)		374.4 (789.2)
Constant	34,532 (3891)***	32,268 (4381)***	39,114 (4141)***	106,650 (149,192)	− 20,664 (151,855)	69,013 (156,651)	− 3163 (156,712)
Observations	603	603	603	603	603	603	603
*R*-squared	0.011	0.076	0.155	0.171	0.209	0.197	0.212

Robust standard errors in parentheses

***p < 0.01, **p < 0.05, *p < 0.1

Notes: ^1^the reference category is land for other uses (e.g. livestock rearing). *q/ha* quintal per hectare, *đ/q* dongs per quintal

OLS estimates; s.e. in brackets

A different interpretation of the negative sign of the coefficient of the quantity of land cultivated is possible. More land also means more money to be spent on seeds, agrochemicals and so on; therefore, the negative coefficient may indicate that farmers prefer to invest more in production than in protection. Consistent with the extant literature, I find no correlation between risk awareness and expenditure on PPE. However, farmers who have been exposed to sensitising campaigns spend more on PPE than the non-exposed. Interestingly, an ancillary ordered probit regression reveals that risk awareness does not correlate with the exposure to sensitising campaigns. This suggests that these programmes stimulate investment in personal protection while not changing the perception of the risks associated with the use of pesticides. The last noteworthy outcome is the negative and statistically significant coefficient of the concentration of sprayed pesticides per hectare of cultivated land. This outcome suggests that the more concentrated the chemical product is, the less the farmers invest in PPE. Again, this may suggest some substitutability between the investments in the productive process and the investments in PPE. Thus, it seems that farmers who buy (and use) more chemicals then lack the money to purchase PPE. I wish to stress that in the regressions, I also control for the number of sprays; therefore, for a given number, higher concentrations entail the need to purchase more agrochemicals with respect to farmers who use less concentrated liquids.

Table 5 reports the estimates for the level of PPE assessed through the first of the two principal components, obtained following the method described in Section 3. Income per capita displays a negative and statistically significant coefficient. This suggests that as the average individual income increases, the level of completeness of the protection used by the farmer decreases. The link between wealth and the level of PPE is positive but weak: Only the fifth component of wealth presents a coefficient that is statistically significant, while the others and the coefficient for the extension of the cultivated land are not significantly different from zero. However, the total expenditure on PPE displays a positive link with the level of PPE.

**Table 5:** Determinants of completeness of PPE (first principal component)

	1	2	3	4	5	6	7	8
Expenditure to purchase PPE (annual, million dongs)	32.47 (2.672)***	33.67 (2.721)***	33.98 (2.779)***	34.20 (2.843)***	34.12 (2.957)***	33.89 (2.902)***	33.98 (2.966)***	
Income per capita (million dongs)		− 0.0385 (0.00833)***	− 0.0365 (0.00850)***	− 0.0368 (0.00874)***	− 0.0373 (0.00861)***	− 0.0385 (0.00881)***	− 0.0383 (0.00874)***	− 0.00596 (0.0107)
Income from crops (% of total household income)		0.00135 (0.00168)	0.00135 (0.00167)	0.00139 (0.00167)	0.00129 (0.00171)	0.00131 (0.00166)	0.00130 (0.00169)	0.00128 (0.00205)
Remittances (dongs)		2.83e-09 (1.76e-08)	4.11e-09 (1.81e-08)	4.07e-09 (1.77e-08)	3.54e-09 (1.74e-08)	3.12e-09 (1.66e-08)	3.37e-09 (1.69e-08)	−1.77e-08 (1.78e-08)
Household’s wealth (principal components)								
Component 1			− 0.00674 (0.0222)	− 0.0114 (0.0228)	− 0.0124 (0.0227)	− 0.0182 (0.0238)	− 0.0179 (0.0238)	− 0.0993 (0.0281)***
Component 2			0.00106 (0.0410)	0.00479 (0.0412)	0.00239 (0.0418)	0.00509 (0.0415)	0.00363 (0.0416)	0.0534 (0.0479)
Component 3			0.0578 (0.0381)	0.0545 (0.0388)	0.0532 (0.0391)	0.0551 (0.0383)	0.0557 (0.0386)	0.0623 (0.0474)
Component 4			− 0.00627 (0.0313)	− 0.00741 (0.0318)	− 0.00634 (0.0318)	− 0.00656 (0.0317)	− 0.00772 (0.0317)	− 0.0228 (0.0407)
Component 5			0.0787 (0.0392)**	0.0739 (0.0399)*	0.0814 (0.0415)*	0.0793 (0.0401)**	0.0789 (0.0418)*	−0.0133 (0.0474)
Extension of cultivated land (ha)	− 0.0185 (0.0293)	− 0.0487 (0.0340)	− 0.0434 (0.0369)	1.648 (0.868)*	1.653 (0.951)*	1.443 (0.996)	1.511 (0.998)	0.196 (1.486)
Number of pesticide applications in the current season	− 0.00776 (0.0282)	− 0.0101 (0.0283)	− 0.00767 (0.0279)	− 0.00841 (0.0279)	− 0.00413 (0.0286)	− 0.00319 (0.0283)	− 0.00321 (0.0285)	0.0273 (0.0332)
Concentration of pesticides sprayed (mg/ha)	7.44e-05 (0.000171)	0.000140 (0.000164)	0.000145 (0.000166)	0.000123 (0.000164)	0.000106 (0.000160)	0.000135 (0.000167)	0.000124 (0.000164)	− 0.000140 (0.000190)
Number of pesticide sprayers		0.270 (0.0898)***	0.276 (0.0900)***	0.287 (0.0924)***	0.291 (0.0932)***	0.288 (0.0925)***	0.291 (0.0932)***	0.261 (0.114)**
Average unit cost of sprayers (dongs)		− 3.78e-07 (2.39e-07)	− 3.59e-07 (2.38e-07)	− 3.64e-07 (2.43e-07)	− 3.65e-07 (2.44e-07)	− 3.54e-07 (2.38e-07)	− 3.53e-07 (2.39e-07)	− 5.41e-07 (2.74e-07)**
Money borrowed (million dongs)		0.00341 (0.00463)	0.00258 (0.00460)	0.00371 (0.00476)	0.00413 (0.00486)	0.00360 (0.00477)	0.00388 (0.00486)	− 0.00392 (0.00582)
Age				0.00242 (0.00362)	0.00247 (0.00371)	0.00235 (0.00386)	0.00251 (0.00391)	0.00299 (0.00499)
Use of land (ha allocated to the following crops/activities)^1^								
Rice				− 1.693 (0.868)*	− 1.692 (0.949)*	− 1.489 (0.995)	− 1.554 (0.995)	− 0.272 (1.484)
Orchards				− 1.943 (0.870)**	− 1.907 (0.939)**	− 1.688 (0.997)*	− 1.767 (0.985)*	− 0.215 (1.469)
Other vegetables				− 1.650 (0.885)*	− 1.753 (0.974)*	− 1.563 (1.020)	− 1.618 (1.020)	− 0.365 (1.507)
Aquaculture				− 0.310 (0.977)	− 0.253 (1.059)	− 0.0500 (1.093)	− 0.111 (1.098)	2.087 (1.681)
Yield of the main crop in the last season (q/ha)					0.0269 (0.0139)*	0.0267 (0.0135)**	0.0269 (0.0140)*	0.0393 (0.0183)**
Yield of the main crop in the second-to-last season (q/ha)					0.00515 (0.00294)*	0.00549 (0.00327)*	0.00534 (0.00338)*	0.00701 (0.00417)*
Price of the main crop in the previous season (đ/q)					− 8.82e-05 (6.60e-05)	− 9.07e-05 (6.60e-05)	− 9.02e-05 (6.59e-05)	− 3.70e-05 (8.22e-05)
Price of the main crop in the second-to-last season (đ/q)					3.84e-05 (6.60e-05)	3.72e-05 (6.70e-05)	4.02e-05 (6.60e-05)	0.000151 (6.02e-05)**
Practices IPM (dummy: 1 = yes)					− 0.0464 (0.107)		− 0.0432 (0.107)	− 0.00489 (0.00468)
Received formal training on IPM (dummy: 1 = yes)					− 0.0283 (0.108)		− 0.0274 (0.108)	0.0228 (0.0415)
Education								
No school						− 0.0495 (0.517)	− 0.0355 (0.512)	− 0.122 (0.615)
Primary school						0.119 (0.131)	0.119 (0.133)	0.169 (0.158)
Secondary school						0.151 (0.126)	0.150 (0.124)	0.251 (0.143)*
Level of risk perceived					0.00723 (0.0352)		0.0100 (0.0354)	0.0799 (0.126)
Constant	− 1.272 (0.192)***	− 1.337 (0.219)***	− 1.385 (0.217)***	− 6.135 (7.125)	− 6.291 (7.280)	− 6.194 (7.600)	− 6.492 (7.696)	− 6.599 (9.837)
Observations	603	603	603	603	603	603	603	603
*R*-squared	0.322	0.351	0.357	0.362	0.367	0.368	0.369	0.090

***p < 0.01, **p < 0.05, *p < 0.1

Notes: ^1^the reference category is land for other uses (e.g. livestock rearing). *q/ha* quintal per hectare, *đ/q* dongs per quintal

Robust standard errors in parentheses

OLS estimates; s.e. in brackets

The interpretation of these results, which leads to the analyses presented in Table 7, is that as the farmers’ income increases, when purchasing protective equipment, they focus on the quality rather than on the completeness of the protection, i.e. they buy more expensive equipment, trading off quality (or perceived quality, assuming that the price of a purchased item increases with the quality of the good) for quantity. Apparently, instead of buying a complete set of protection (i.e. trousers, shirts, gloves, masks, glasses), farmers buy selected items (e.g. trousers and glasses) with a higher price (and likely better quality). This behaviour is not necessarily bad, as, if the purchased goods are really of better quality, they protect the worker better than analogous goods of poorer quality. However, this result highlights that the money invested in PPE is in any case less than the amount that is needed to ensure full and adequate protection. The last column of the table shows the result of a specification that excludes expenditure on PPE, as it correlates positively with per capita income (correlation coefficient: 0.217, *p* value < 0.01). The figures in the column suggest that PPE expenditure is a relevant variable whose omission from the specification biases the results. Indeed, when it is excluded, part of its effect is captured by income per capita, which, however, correlates positively with it and negatively with the dependent variable. Given the magnitude of the coefficients, the result is pushing the coefficient of per capita income close to zero while increasing its standard error (which is now affected also by the variance of the correlated omitted variable). Consequently, the specifications that include both the regressors are to prefer.

An interesting result is that the yields in the two previous crop seasons show a positive and statistically significant effect on the level of protection. This seems to suggest that part of the income of a good crop season is invested in PPE.

Table 6 reports the OLS estimates for the level of PPE, assessed through the second of the two principal components, obtained in the way described in Section 3. The results are analogous to those presented in the previous table. I should recall here that the difference between these two components lies in the fact that the first also correlates positively with the use of shirts and trousers, while the second shows a negative link with these two protective garments. The strong similarity between the results shown in Tables 5 and 6 and the differences between the two components suggests that the purchase of trousers and shirts on the one side and the purchase of other forms of protection on the other side follow different decisional paths. Actually, shirts and trousers may also be used during work other than pesticide usage, while masks, glasses and gloves have a more specific—and thus limited—purpose. These considerations justify the analysis of the expenditure on each single form of protection listed by the survey (which contains seven such protective tools). For the same reasons as those explained before, the eighth specification presented in the table excludes expenditure on PPE from the set of regressors. The same comments as before hold also in this case.

**Table 6:** Determinants of completeness of PPE (second principal component)

	1	2	3	4	5	6	7	8
Expenditure to purchase PPE (annual, million dongs)	13.43 (2.692)***	15.41 (2.739)***	16.57 (2.782)***	16.70 (2.822)***	15.50 (2.949)***	15.84 (2.886)***	15.53 (2.974)***	
Income per capita (million dongs)		− 0.0321 (0.00866)***	− 0.0300 (0.00886)***	− 0.0297 (0.00900)***	− 0.0309 (0.00884)***	− 0.0310 (0.00915)***	− 0.0315 (0.00904)***	− 0.0167 (0.00986)*
Income from crops (% of total household income)		0.00181 (0.00181)	0.00173 (0.00181)	0.00170 (0.00182)	0.00162 (0.00183)	0.00150 (0.00178)	0.00149 (0.00181)	0.00148 (0.00183)
Remittances (dongs)		− 1.21e-08 (1.87e-08)	− 2.60e-09 (2.18e-08)	− 7.87e-10 (2.17e-08)	− 4.20e-09 (1.95e-08)	− 4.15e-09 (1.99e-08)	− 3.94e-09 (1.94e-08)	− 1.36e-08 (1.80e-08)
Household’s wealth (principal components)								
Component 1			0.0378 (0.0221)*	0.0341 (0.0230)	0.0446 (0.0229)*	0.0514 (0.0237)**	0.0519 (0.0237)**	0.0147 (0.0256)
Component 2			− 0.0300 (0.0435)	− 0.0307 (0.0440)	− 0.0288 (0.0443)	− 0.0278 (0.0449)	− 0.0286 (0.0445)	− 0.00592 (0.0449)
Component 3			0.0695 (0.0401)*	0.0685 (0.0407)*	0.0587 (0.0408)	0.0637 (0.0399)	0.0591 (0.0398)	0.0621 (0.0430)
Component 4			0.0227 (0.0317)	0.0236 (0.0317)	0.0267 (0.0328)	0.0206 (0.0323)	0.0245 (0.0331)	0.0176 (0.0347)
Component 5			0.0597 (0.0413)	0.0570 (0.0413)	0.0607 (0.0437)	0.0681 (0.0414)	0.0635 (0.0434)	0.0214 (0.0459)
Extension of cultivated land (ha)	− 0.0240 (0.0307)	− 0.0642 (0.0345)*	− 0.0428 (0.0367)	− 1.069 (1.072)	− 1.297 (1.244)	− 1.501 (1.159)	− 1.418 (1.261)	− 2.019 (1.474)
Number of pesticide applications in the current season	− 0.00768 (0.0291)	− 0.00463 (0.0295)	− 0.000170 (0.0295)	− 0.000710 (0.0296)	0.00184 (0.0307)	0.00108 (0.0302)	0.00335 (0.0306)	0.0173 (0.0318)
Concentration of pesticides sprayed (mg/ha)	− 0.000615 (0.000190)***	− 0.000511 (0.000193)***	− 0.000483 (0.000200)**	− 0.000499 (0.000200)**	− 0.000476 (0.000193)**	− 0.000506 (0.000205)**	− 0.000478 (0.000197)**	− 0.000598 (0.000197)***
Number of pesticide sprayers		0.181 (0.0953)*	0.195 (0.0943)**	0.196 (0.0953)**	0.185 (0.0967)*	0.205 (0.0952)**	0.192 (0.0969)**	0.179 (0.102)*
Average unit cost of sprayers (dongs)		2.15e-07 (2.46e-07)	2.34e-07 (2.42e-07)	2.29e-07 (2.43e-07)	2.25e-07 (2.44e-07)	2.27e-07 (2.30e-07)	2.29e-07 (2.37e-07)	1.43e-07 (2.28e-07)
Money borrowed (million dongs)		0.00343 (0.00459)	0.00337 (0.00466)	0.00384 (0.00480)	0.00307 (0.00495)	0.00335 (0.00474)	0.00263 (0.00489)	− 0.000933 (0.00492)
Age				0.00460 (0.00388)	0.00542 (0.00407)	0.00362 (0.00404)	0.00353 (0.00415)	0.00375 (0.00442)
Use of land (ha allocated to the following crops/activities)^1^								
Rice				1.021 (1.070)	1.247 (1.240)	1.460 (1.156)	1.371 (1.257)	1.957 (1.472)
Orchards				0.964 (1.075)	1.195 (1.234)	1.419 (1.160)	1.339 (1.251)	2.049 (1.469)
Other vegetables				1.184 (1.094)	1.473 (1.266)	1.722 (1.188)	1.618 (1.284)	2.191 (1.494)
Aquaculture				1.083 (1.232)	1.291 (1.392)	1.614 (1.329)	1.477 (1.419)	2.481 (1.688)
Yield of the main crop in the last season (q/ha)					0.0175 (0.0134)	0.0175 (0.0131)	0.0165 (0.0131)	0.0222 (0.0149)
Yield of the main crop in the second-to-last season (q/ha)					− 0.00110 (0.00283)	− 0.00368 (0.00304)	− 0.00305 (0.00321)	− 0.00229 (0.00359)
Price of the main crop in the previous season (đ/q)					6.27e-06 (6.70e-05)	− 3.54e-06 (6.66e-05)	7.88e-07 (6.60e-05)	2.51e-05 (6.84e-05)
Price of the main crop in the second-to-last season (đ/q)					0.000141 (5.37e-05)***	0.000139 (5.53e-05)**	0.000133 (5.66e-05)**	0.000183 (5.30e-05)***
Practices IPM (dummy: 1 = yes)					0.0261 (0.113)		0.0317 (0.113)	0.0149 (0.0398)
Received formal training on IPM (dummy: 1 = yes)					0.161 (0.115)		0.137 (0.113)	0.186 (0.111)*
Education								
No school						0.600 (0.676)	0.540 (0.668)	0.500 (0.626)
Primary school						− 0.181 (0.140)	− 0.170 (0.143)	− 0.147 (0.149)
Secondary school						− 0.000563 (0.139)	0.00333 (0.134)	0.0497 (0.141)
Level of risk perceived					0.0145 (0.0380)		0.00913 (0.0381)	0.0826 (0.116)
Constant	− 0.434 (0.200)**	− 0.712 (0.242)***	− 0.839 (0.245)***	− 9.857 (7.630)	− 11.82 (7.981)	− 8.084 (7.969)	− 8.021 (8.161)	− 8.070 (8.678)
Observations	603	603	603	603	603	603	603	603
*R*-squared	0.079	0.112	0.122	0.125	0.140	0.143	0.147	0.076

Robust standard errors in parentheses

***p < 0.01, **p < 0.05, *p < 0.1

Notes: ^1^the reference category is land for other uses (e.g. livestock rearing). *q/ha* quintal per hectare, *đ/q* dongs per quintal

OLS estimates; s.e. in brackets

For the sake of conciseness, although as before several specifications are estimated, Table 7 reports the results only for the specification that includes the most variables. Moreover, only the coefficients of the main variables of interest are shown.

**Table 7:** Determinants of the expenditure for each protection; OLS estimates, s.e. in brackets^1^

	Shoes	Hat	Glasses	Mask	Shirt	Trousers	Gloves
Expenditure to purchase PPE (annual, dongs)	0.0136 (0.00709)*	0.237 (0.0279)***	0.145 (0.0252)***	0.0438 (0.0132)***	0.151 (0.0152)***	0.218 (0.0201)***	0.0150 (0.00306)***
Income per capita (million dongs)	− 27.01 (12.72)**	− 242.4 (80.72)***	− 19.92 (108.0)	− 3.085 (21.83)	201.3 (63.41)***	283.1 (66.65)***	− 47.40 (15.19)***
Income from crops (% of total household income)	− 0.518 (1.613)	− 18.55 (11.66)	− 0.404 (9.194)	1.524 (3.046)	1.702 (7.177)	4.303 (9.156)	1.020 (1.438)
Remittances (dongs)	1.28e-05 (1.28e-05)	0.000111 (0.000140)	− 1.39e-05 (5.81e-05)	− 1.50e-05 (2.80e-05)	7.69e-05 (5.98e-05)	− 2.77e-05 (0.000106)	− 2.18e-05 (1.46e-05)
Household’s wealth (principal components)							
Component 1	22.02 (24.98)	487.9 (216.5)**	− 30.88 (179.7)	26.10 (42.42)	− 182.8 (121.2)	− 338.7 (159.2)**	11.05 (24.68)
Component 2	− 56.81 (44.59)	− 64.33 (299.2)	158.6 (221.7)	− 4.055 (92.22)	377.1 (168.6)**	636.2 (205.1)***	5.196 (43.83)
Component 3	− 19.67 (70.84)	− 100.1 (285.4)	561.5 (257.3)**	− 115.0 (127.0)	26.67 (178.3)	− 286.0 (247.9)	18.16 (49.91)
Component 4	5.444 (32.17)	− 189.6 (347.1)	− 125.0 (170.7)	− 53.02 (83.90)	− 214.8 (158.7)	− 12.33 (253.1)	57.73 (35.79)
Component 5	160.7 (75.53)**	− 123.3 (354.4)	405.5 (306.2)	− 107.0 (78.38)	− 656.5 (201.1)***	− 940.3 (251.3)***	− 58.19 (47.15)
Extension of cultivated land (ha)	− 1110 (840.1)	− 5755 (3304)*	− 960.1 (2491)	1515 (1344)	2819 (4063)	3804 (3647)	2362 (1375)*
Duration of the good (weeks)	559.7 (126.7)***	378.7 (57.09)***	498.1 (83.68)***	373.5 (68.53)***	127.8 (49.29)***	469.9 (57.34)***	1002 (109.0)***
Level of risk perceived	− 69.55 (40.93)*	744.7 (323.8)**	− 291.6 (226.0)	−1 83.8 (81.46)**	− 262.6 (160.0)	− 396.6 (191.8)**	− 16.21 (30.09)
Constant	− 18,789 (7282)**	− 10,656 (52,636)	− 82,651 (44,864)*	− 8691 (14,487)	44,813 (38,532)	74,300 (45,350)	417.4 (7862)
Observations	603	603	603	603	603	603	603
*R*-squared	0.513	0.541	0.542	0.342	0.477	0.625	0.601

***p < 0.01, **p < 0.05, *p < 0.1

Notes: ^1^The figures in the table are estimated using the same seventh specification as in Tables 5 and 6. For the sake of conciseness, only the most relevant variables are reported

The evidence provides about the correlation between per capita income and expenditure on PPE may suggest that other relevant variables are omitted, so engendering problems of endogeneity. To test for this possibility, GMM IV regressions were run and are presented in Tables A1 and A2 in Appendix 1. The results of Durbin-Wu-Hausman tests suggest that endogeneity does not affect the regressions presented in Tables 5 and 6. Further comments on Tables A1 and A2 are available in Appendix 1.

The results of the last part of the analysis presented in this paper confirm the previous interpretation. Indeed, as the per capita income increases, the expenditure on shirts and trousers also increases, while the interviewed farmers reduce their expenditure on many other protective items as the per capita income increases. However, this outcome is to be interpreted together with the positive and statistically significant coefficient of the total amount spent on PPE. The two effects together suggest that as the money spent on PPE increases, so does the expenditure on each single item included in the list of forms of protection. However, the expenditure on shirts and trousers grows more than proportionally with respect to the per capita income, while the opposite happens for the rest of the equipment: The demand for shirts and trousers with respect to the total income is more elastic than that for the other forms of protection.

In conclusion, the results presented in this paper suggest that the inadequateness of the PPE used by the farmers of the Mekong Delta is not (only) a matter of unaffordability but rather a result of the households deciding not to invest in adequate PPE, diverting money from this to other purposes (likely to expand and improve the production). The only two goods for which the expenditure grows more than proportionally with respect to the income per capita are shirts and trousers. However, these garments are of use in activities besides the application of pesticides. Perhaps, after washing, they may also be used outside the working duties.

It might be useful to discuss the results in the light of those obtained by similar studies in other countries worldwide. The main researches on Vietnam are already summarised in the first section of the paper. The empirical outcomes shown in the present paper substantially confirm that Vietnamese farmers underuse PPE. Moreover, the results allow to understand that socio-economic status is a major factor explaining such a behaviour, while insufficient education and awareness may also explain why PPE is underused in the Mekong Delta. The extant literature shows that insufficient personal protection is common in most of the developing countries, also in those, as Argentina, characterised by middle-high levels of income (Aiassa et al. 2019). Such an underuse is common to workers even when they perceive the risks and the damages that the lack of PPE may cause to them. Borges-Khayat et al. (2013) use Brazilian data to show that the workers who use inadequate PPE are more exposed than those who use them to genetic damages engendered by the absorption of agrochemicals. Aiassa et al. (2019) and Balderrama-Carmona et al. (2019) respectively highlight that Argentinian workers in Córdoba and Mexican farmers in Sonora inhale toxic quantities of herbicides, as they underuse PPE, even if many of the consequences of such a behaviour are clear to the farmers. The situation seems particularly bad in poorer countries, where the largest share (80%) of casualties for agrochemical poisoning occur and only a minority of workers use adequate PPE (Chitra et al. 2006; Barrón-Cuenca et al. 2020). Sapbamrer and Thammachai (2020) present a review of an extensive number of studies; from there, a clear picture that farmers in more developed countries use more PPE than farmers in poorer countries emerges. Agricultural workers in Indonesia, South Korea and Thailand use more PPE than their colleagues in Cambodia, Nepal and the Philippines. Moving to PPE is found to be more used and adequate in Ghana and Egypt than in Ethiopia and Zimbabwe.^4^ However, also in developed countries, the use of complete PPE is not common to all the agricultural workers; for example, Greek tobacco growers are found to underuse PPE (Damalas et al. 2006), although the lack of protection appears less severe here than in developing countries. Some authors (for example Reddy et al. 2016; Barrón-Cuenca et al. 2020) suggest to train farmers and workers to increase the use of PPE. Nevertheless, Migheli (2020) suggests that also trained farmers in Vietnam are underusing PPE, although training has some positive impact on it. Such a result indicates that the causes of inadequate protections should be searched also elsewhere than in the absence of training. A major candidate to be an explanatory factor is the socio-economic status of the farmers, as suggested in the first section of the paper. Some studies have inquired the relationship between income of farmers and use of PPE, with mixed results. On the one hand, there are studies that did not find any such relationship: Okoffo et al. (2016) consider cocoa growers in Ghana and show that income from cocoa and use of PPE are not correlated; similar results are also found in a sample of cocoa farmers in Cameroon (Oyekale 2018). On the other hand, a recent review (Sapbamrer and Thammachai 2020) finds general evidence that socio-economic status of farmers and agricultural workers is positively associated with the use of PPE and that the higher the income, the more complete the PPE used is. From this review, what emerges is that low-income countries provide their farmers with levels of income that are too low to allow them to purchase a minimum of PPE, so explaining the results found in Ghana and Cameroon. However, as the general socio-economic situation improves, farmers are more and more able to use part of their income to buy PPE. Therefore, the underuse of it is less severe in middle- and high-income countries. Vietnam falls in the group of middle-income countries; therefore, the results presented in this paper are in line with the empirical evidence found around the world by the extant literature. The evidence provided in the present paper adds to the existing literature, as unveils the relationship between socio-economic factors and use of PPE in a fast-growing, yet still developing, country, where agriculture represents a major source of income for a large share of the population. The results from more advanced countries allow hoping that the currently non-good situation in Vietnam will improve thanks to the economic development of the country.

Appendix 2 presents some evidence that substitution effects exist between risk awareness and income per capita in determining PPE completeness.

## Discussion and limitations

While the results of this study are partial, as they refer to a specific region of the world, they add more evidence to what seems to be a widespread phenomenon: underinvestment in PPE. The evidence presented in this paper suggests that farmers in the Mekong Delta could invest more resources in PPE, but they do not. The lack of adequate protection increases the probability of incurring acute and chronic intoxication. The figures presented in the tables suggest that:

- The economic conditions of farmer households are very relevant for the level of protection used when applying pesticides.
- Not only income, as one would easily expect, correlates positively and significantly with expenditure on PPE, but also the household wealth—at least as measured here—plays a role.
- A worrying result that emerges from estimates is that the more pesticides the farmers use, the less they invest on PPE for given levels of income and wealth.

A major reason may be that farmers in developing countries struggle to increase their income and therefore concentrate their investments in production rather than in PPE, regardless of the risks entailed by the use of pesticides and of agrochemicals in general.

On the one side, the third result highlighted in the list may reflect some substitution between PPE and the purchase of agrochemicals: As the economic resources are limited, farmers may prefer to buy pesticides rather than protective equipment to increase the yield of their crops at the price of risking health. Such a strategy may be understandable, as poisoning may result from the accumulation of chemical agents in the farmers’ body in the long run. This evidence suggests that the users of pesticides may not understand the consequences of under-protection immediately: (hoped) increases in crop yields may come before than health problems. On the other side, lack of knowledge about how to manage the products used and how to protect themselves adequately play—according to the literature—a very relevant role.

Whatever the reason for Vietnamese farmers’ underinvestment in PPE, the government and the international institutions in this country should intervene to convince the farmers to purchase adequate PPE and to use it. Besides the decrease in the financial burden for the national health system, which has to care for intoxicated workers, improvements in the quality life of the latter are also a likely outcome of further investments in PPE. The current programmes that pursue this aim are indeed either ineffective or insufficient to reach the goal.

In addition, programmes aimed at increasing the adoption of integrated pest management should be reinforced, as the substitution of chemical products with natural remedies (such as natural antagonists of pests) may reduce the risk of poisoning without requiring increasing in the income and wealth of farmers’ households. These last may indeed be difficult to achieve in the short run, as the adoption of new and more efficient technologies of cultivation requires financial efforts and time. In addition, the volatility that characterises the prices of agricultural products may constitute an additional problem.

Further research should aim to provide an understanding of the main reasons that push farmers to under-protect themselves. This research should be instrumental in the design of policies that are effective in promoting the purchase and the use of PPE.

The main limits of the analysis presented in the paper are represented by the area under consideration and the specific economic conditions of the country where it is located. On the one side, the cultivation of rice characterises the Mekong Delta; such a crop may require treatments that are different from other crops, rendering the results of the present study non-generalizable to other cases. In addition, the peculiar economic and political situation of Vietnam are not common to other countries, characterised by higher or lower incomes per capita and less public intervention in training and education; consequently, different conditions may lead to different farmers’ behaviours. Another limit is due to the lack of data on how informed the farmers included in the sample are about the risks deriving from handling pesticides and about the adequate protections to be used.

## Conclusions

The empirical analysis presented in the paper highlights that the socio-economic conditions of farmers, at least in the Mekong Delta, are a major cause of insufficient protection when agrochemicals are used. This evidence implies that as often observed in the case of developing countries, the lack of education and training is not sufficient to explain why farmers under-protect themselves. Therefore, programs that aim at increasing the use of PPE should consider also the lack of economic resources as a major factor that affects negatively their success. The empirical evidence presented in Appendix 2 shows the existence of some substitutability between income and risk perception in the adoption of complete PPE. Such a result may suggest designing policies that aim at increasing farmers’ risk awareness could be as useful as helping them to increase their income.

## Supplementary information

ESM 1 (DOCX 39 kb)

## Data Availability

The data are available in the World Bank Microdata Repository at the link indicated in footnote 3.

## Footnotes

1 Eddleston (2020), p. 214

2 This is a relatively large number for this type of study.

3 http://archive.iwlearn.net/mrcmekong.org/ISH/SEA-Baseline/13.%20Vietnam%20Baseline%20Assessment%20Perspective%2028%20Jan.pdf

4 The authors assign Jamaica to Africa.
