# Supplementary material for: Income, wealth and use of personal protection equipment in the Mekong Delta
Source: Environ Sci Pollut Res Int. 2021 Mar 25;28(29):39920–37. doi: 10.1007/s11356-021-13449-w (PMC8310476; doi:10.1007/s11356-021-13449-w)
Supplement: Supplementary file 1 — (DOCX 39 kb) [file 11356_2021_13449_MOESM1_ESM.docx]

**Appendix 1. GMM IV estimates**

The following tables report GMM IV estimates for six specifications of the model, whose estimates are presented in Tables 5 and 6 of the paper. The instrumented variable is per capita income, which may be suspected of endogeneity. Given the limited amount of variables contained in the dataset, instruments were chosen partially among the regressors included in some of the specifications presented in Tables 5 and 6 and this explains why the specifications presented in the following tables partially differ from those in the tables included in the main body of the paper.

The variables used as instruments are: the quantities of crops sold during the previous season, the level of education of the respondent, the extension of owned cultivated land, the extension of land used for residential buildings and the fourth principal component of household wealth – the only one among the five components, whose correlation with income is statistically significant). Looking at Tables 5 and 6 allows to see that the variables included in the specifications presented in those tables and used as instruments are not significantly correlated to the dependent variable. Therefore they can be removed from the set of regressors and used as instruments.

The following tables report the estimates for these regressions. It is possible to see that income per capita retains its sign and some statistical significance only in the fifth and sixth specification presented in Table A1; no statistically significant effect is present in Table A2 with reference to income per capita. Nevertheless, it is noteworthy that the sign of the coefficients of the instrumented variable is consistent with that that the same variable has in Tables 5 and 6. Two caveats are in order here: on the one hand, as the Durbin-Wu-Hausman tests suggest, there is no need for instrumenting income per capita in these regressions. On the other hand, Stock-Yogo tests on the weakness of the instruments suggest that the instruments used in the regressions are often weak, i.e. while they are correlated with the instrumented variable and uncorrelated with the dependent variable, their correlation with the first is generally low, though statistically significant. The combination of these two pieces of evidence (i.e. no need for instrumenting and weakness of some instruments) suggest to preserve the results shown in Tables 5 and 6 in the main text.

.

**Appendix 2. Interaction effects between income per capita and risk awareness^[[1]](#footnote-1)^**

The following table reports the coefficients of per capita income, risk awareness and their interactions, considering regressions that include the same variables as those used in the seventh specification presented in Tables 5 and 6. The figures show that: as risk perception increases so does PPE completeness as measured by the variables used in the paper. Overall the contribution of income remains negative, although, the contribution to PPE completeness once cleaned by the interaction with risk perception, is now positive, reinforcing the idea that, if farmers had more income, they would spend more on PPE. Indeed, the coefficient for the per capita income in these regressions represents a pure income effect, cleaned by the substitution effect between risk perception and income. The negative sign presented by the coefficients of the interacted variables suggests that the income per capita and risk perception are substitutes in determining the level of PPE completeness. In sum, the degree of PPE completeness depends on either the level of riskiness perceived or on income per capita. The substitutability detected between risk perception and income suggests that policies aimed at increasing risk awareness and policies targeted at increasing farmers’ income would be effective in increasing the level of protection used.

1. The author is thankful to an anonymous referee, who suggested this additional piece of analysis. [↑](#footnote-ref-1)
